# Assessment of the Anti-Hyperglycaemic, Anti-Inflammatory and Antioxidant Activities of the Methanol Extract of *Moringa Oleifera* in Diabetes-Induced Nephrotoxic Male Wistar Rats

**DOI:** 10.3390/molecules22040439

**Published:** 2017-03-23

**Authors:** Elizabeth I. Omodanisi, Yapo G. Aboua, Oluwafemi O. Oguntibeju

**Affiliations:** 1 Phytomedicine and Diabetes Research Group, Oxidative Stress Research Centre, Department of Biomedical Sciences, Faculty of Health and Wellness Sciences, Cape Peninsula University of Technology, P.O. Box 1906, Bellville 7535, South Africa; lizzy.omodanisi@gmail.com; 2 Faculty of Health and Wellness Sciences, Cape Peninsula University of Technology, P.O. Box 1906, Bellville 7535, South Africa; abouay@cput.ac.za

**Keywords:** *Moringa oleifera*, diabetes, methanolic extract, streptozotocin, hyperglycemia, nephrotoxicity, phytochemical, antioxidant, flavonoids, inflammatory

## Abstract

Diabetes mellitus is an endocrine disease of multiple aetiologies in insulin secretion. A deficiency in insulin results in hyperglycemia with metabolic disturbances of biomolecules. *Moringa oleifera* (MO) is endemic in the tropics with a variety of ethnomedicinal importance. The leaf of this plant has been reported to possess antioxidant and medicinal properties that may be helpful in the treatment and management of diabetes and its associated complications. Diabetes was induced intraperitoneally in rats by a single dose of streptozotocin (55 mg/kg) and treated with methanolic extract of *Moringa oleifera* (250 mg/kg b.wt) for six weeks. Forty-eight (48) adult male Wistar strain rats were randomly divided into four groups: normal control (NC), *Moringa oleifera* treated control rats (NC + MO), diabetic rats (DM) and *Moringa oleifera* treated diabetic rats (DM + MO). Estimation of antioxidant capacity, total polyphenols, flavonoids and flavonols content of *Moringa oleifera* extract was performed and serum biochemical markers were evaluated. Antioxidants such as catalase (CAT), glutathione peroxidase (GPx), superoxide dismutase (SOD) activities, glutathione (GSH) and inflammatory biomarkers were determined in the kidney. Results showed high antioxidant capacities of MO extract and improved serum biochemical markers, whilst lipid peroxidation (MDA) levels were reduced in non-diabetic and diabetic rats after MO treatment when compared to normal control. Subsequent administration of MO led to an increased concentration of serum albumin, globulin and total protein with a decrease in the level of MDA, and improvements in CAT, SOD, GSH, GPx, (tumour necrosis factor-alpha)TNF-α and (interleukin-6)IL-6. MO contains potent phytochemical constituents that offer protective action against diabetic-induced renal damage, reactive oxygen species (ROS) and inflammation and could therefore play a role in reducing diabetic complications, particularly in developing countries such as in Africa where the majority cannot afford orthodox medicine.

## 1. Introduction

The increasing prevalence of diabetes in both developed and developing countries has challenged scientists to further conduct research in sourcing for potent therapeutic agents from natural sources for more efficient usage in the treatment and management of diabetes [1]. There is an emerging global epidemic of diabetes that can be traced back to rapid increase in weight, obesity and sedentary lifestyle [2]. Diabetes is a degenerative disease of the blood glucose system, characterized by pancreatic beta cells’ deficiency to produce insulin or sufficient insulin, resulting in chronic hyperglycemia, which is associated with long-term microvascular (retinopathy, nephropathy, and neuropathy) and macrovascular (cardiovascular) complications [2]. Hyperglycemia-induced oxidative stress is implicated in the onset and progression of diabetes and, if left untreated, can lead to severe complications [3]. Diabetes is predicted to become the seventh leading cause of death in the world by 2030 and total deaths from diabetes are projected to rise by more than 50% in the next 10 years [4].

Individuals with diabetes have high levels of inflammatory cytokines, activation of leukocytes and increased tissue fibrosis [5]. Accumulation of ROS leads to oxidative stress, which is associated with increased damage to β-cells and biomolecules [6]. Reactive species are highly reactive atoms with unpaired electrons in their outer orbitals such as superoxide anions, singlet oxygen, hydroxyl radicals, peroxyl radicals and carboxyl radicals [7,8]. ROS are produced by aerobic metabolism, electron transport activity (which releases unpaired electrons), by-products of normal enzymatic reactions, as well as during inflammatory response, stress, and human activities including pollution, alcohol consumption and drugs [9].

Potential damage to biological molecules, cell membranes, cellular lipids, inflammation, β-cells destruction, and eventually cell death are mediated by ROS through direct reaction [10,11]. Unfortunately, over-production of ROS have been implicated in causing extremely harmful effects such as nephropathy, neuropathy, ketoacidosis, retinopathy, cancer, arthritis, coronary heart disease, damage to DNA and proteins (leading to errors in replication), peroxidation of lipids in the blood stream, heart disease and early aging [12,13,14]. Furthermore, over-accumulation of ROS disrupts the antioxidant mechanism, giving rise to a cascade of deleterious events, thereby inactivating antioxidant enzymes and stimulating glycation of proteins, resulting in diabetic complications [15,16].

Given that increased levels of free radicals such as ROS, inflammatory mediators and apoptotic proteins are associated with diabetes mellitus, scavenging activities of MO can help to modulate inflammatory molecules in diabetic rats, protecting tissues from oxidative stress and preventing the development of diabetic complications [17,18]. Previous studies have shown that aqueous, methanolic and ethanolic extracts of MO leaves possess a wide range of biological activities such as antioxidant, antiulcer, analgesic, radio-protective, antihypertensive, and immunomodulatory actions in vivo [19,20]. Researchers have reported hypoglycemic and anti-inflammatory effects of MO in normal and diabetic male rats and showed the ability of MO to lower blood glucose levels and reduce free radical activity [21,22,23,24].

Lipid peroxidation results from free radical chain reaction, causing deterioration in the lipid of the cell membrane and affecting the structural and physiological integrity of cells. This is seen in diabetic conditions, kidney diseases and disruption in blood vessels [25,26]. Antioxidants (non-enzymatic and enzymatic antioxidants) help to mop up free radicals and ROS by preventing lipid peroxidation, thereby reducing deleterious effects caused by these species [27,28,29].

*Moringa oleifera* (MO) has rich antioxidant content and diverse therapeutic abilities. Previous investigation identified MO with the ability to prevent the occurrence and complications of diabetic-induced kidney injury through its protective effect on the oxidative status and inflammatory cytokines in the kidneys of diabetic rats [30]. This plant has been reported to have some analgesic, anti-diabetic, antispasmodic, diuretic, antihypertensive, cholesterol lowering, antioxidant, antibacterial properties and plays beneficial roles in modern medicine [31,32,33].

Remarkably, studies with animal models on markers of oxidative stress and correlation with antioxidant properties in vivo and in vitro systems have been reported, though not extensively. In vivo studies revealed antioxidant capacity of the aqueous extract of MO leaf to possess the potency of increasing the antioxidant status and reduce lipid peroxidation in a dose-dependent manner, while in vitro demonstrated high antioxidant capacity, thereby showing protective effects against ROS [20,34]. Edoga and others reported anti-diabetic and hypotensive activities of MO in albino rats, as the plant played a role as a hypoglycemic agent in lowering blood glucose levels and preventing further cellular damage [35].

Previous studies on the extracts of MO using chromatographic and spectroscopic techniques revealed the presence of notable phenolic compounds such as kaempferol, quercetin, catechin, Gallic acid, caffeic acid, p-coumaric acid, vanillin, ferulic acid, protocatechuic acid, cinnamic acid and epicatechin [17,36]. These secondary metabolites identified from MO extract have been linked to various biological profiles including antioxidant, anti-tuberculosis, analgesic, anticancer, anti-diabetic, antispasmodic, diuretic, antihypertensive, cholesterol lowering, antioxidant, antibacterial and antimicrobial and antimalarial activities exhibited by this plant [36].

Some histological examination of the pancreatic section of diabetic rats revealed degenerative changes in β-cells, which were significantly reversed after treatment with extract of MO [1,37]. Anti-hepatotoxic effect of *Moringa oleifera* and *Vernonia amygdalina* (VA) extracts in streptozotocin (STZ) induced diabetic rats revealed that single and combined extracts of MO and VA have hepato-protective effects and may be effective in reducing liver damage [38].

Our study was planned to examine the anti-hyperglycaemic, anti-inflammatory and anti-oxidative effects of MO in male Wistar rats. This study links diabetes with increased lipid peroxidation and oxidative damage. Antioxidant capacity status of MO has not been extensively estimated, and specific phytochemicals in MO methanolic leaf extract have not been comprehensively evaluated. These were explored in our study with the overarching aim of clarifying the potential of *Moringa oleifera* as a food supplement and bringing to the fore its stunning capabilities that could feasibly revolutionalise pharmacological products in the treatment and management of diabetes.

Furthermore, due to its numerous scientific and health claims, it is appropriate to further investigate the potential treatment regime of *Moringa oleifera* on diabetes in STZ-induced diabetic animal models that can be used to advance clinical techniques on its ameliorative activities. It is anticipated that the outcome will support the ethnomedicinal information of this plant, especially among rural communities.

## 2. Results and Discussion

The rapid discovery of various medicinal plants and natural products with anti-diabetic potentials has provided a remarkable intervention in the history of many diseases including diabetes [39]. The basis for the use of a number of plants as novel remedies for diabetic complications cannot be overemphasized [40,41].

Hyperglycemia-induced oxidative stress has been shown to be actively involved in the onset and progression of diabetes, leading to various complications such as cardiovascular diseases, nephropathy, amputation of limbs and blindness [42,43,44]. The mechanism of STZ (C_8_H_15_N_3_O_7_) as a toxicant used to induce hyperglycemia in experimental animals involves its toxic effect on the beta cells of the pancreatic islet [45]. Consequently, ROS are formed during this process and a cascade of reactions occur leading to increased levels of superoxide radicals, hydrogen peroxide, and hydroxyl radicals with potential damaging effects on cell macromolecules in the animals [30,46].

### 2.1. Effect of Moringa oleifera on Kidney Weight, Relative Kidney Weight and Plasma Glucose Levels of Rats

The effect of MO on kidney weight, relative kidney weight and blood glucose levels of rats is shown in Table 1. The difference in kidney weight between non-diabetic treated rats (NC + MO) and normal control is not significant. However, *Moringa oleifera*-treated control rats (NC + MO) showed a significant (*p* < 0.05) decrease when compared to the diabetic control (DM). Kidney weights of diabetic control rats increased significantly (*p* < 0.05) when compared to normal control (NC). After treatment of diabetic rats (DM + MO) with MO, a significant (*p* < 0.05) decrease was observed when compared to diabetic control (DM). Similar results were observed in the relative kidney weight. Elevated blood glucose level was observed in the diabetic group when compared to normal control. Plasma glucose level decreased significantly (*p* < 0.05) in diabetic rats after treatment when compared to diabetic controls (DM).

Diabetic rats injected with STZ showed elevated plasma glucose levels, which is indicative of hyperglycemia, an observation also reported by other authors [47,48]. However, treatment of rats with MO showed a significant decreased glucose level when compared to diabetic control (Table 1). This implies that *Moringa oleifera* is able to increase the ability of insulin to lower plasma glucose, suggesting its anti-diabetic activity. These results are consistent with other studies [49,50].

Increased kidney size is a sign of acute inflammation and was observed in diabetic rats when compared to normal controls (Table 1). This study agrees with the findings of previous authors who reported that kidney enlargement may be due to hyperplasia (rapid production of the cell leading to enlarged tissues) and hypertrophy (enlargement of cell components) of the kidney [51,52,53]. Treatment with MO reduced kidney size gained, showing a hypolipidemic effect of MO in the kidneys of diabetic rats. Promotion of excessive oxidative stress in the vascular and cellular milieu results in endothelial cell dysfunction, which is one of the earliest and most pivotal metabolic consequences of chronic hyperglycemia [54].

### 2.2. Estimation of Antioxidant Capacity, Total Polyphenols, Flavonoids and Flavonols Content of Moringa oleifera Extracts

Although other studies have been conducted on MO, the antioxidant activity of the methanolic leaf extract has been reported only to a limited extent. Our study expanded upon this in a comprehensive antioxidant study (Table 2).

The assessment of total antioxidant capacities of methanolic extracts of *Moringa oleifera* was conducted using three complementary assays: oxygen radical absorbance capacity (ORAC), Ferric reducing antioxidant power (FRAP), and Trolox equivalence antioxidant capacity (TEAC). The results were estimated as Trolox equivalent per gram as ORAC (3652.14 ± 113.32) µmol TE/L, and TEAC (96.09 ± 1.58) μmol TE/L ascorbic acid equivalent per gram as FRAP (1736 ± 3.08) AAE/L. Results showed high antioxidant capacity of MO to combat reactive species.

In addition, high concentration of total polyphenols, flavonoids and flavonols content were estimated in the methanolic extracts of *Moringa oleifera*. Total polyphenols content of methanolic extract was (2454.00 ± 17.54) mg GAE/L, flavonoids (297.23 ± 30.00) mg CE/L and flavonols (148.70 ± 4.00) mg QE/L.

Antioxidants help the biological system to defend itself, mop up and repair damages caused by free radicals, and this is done by donating an electron to free radicals to make them stable [38]. Phytochemicals such as polyphenols, flavonoids, flavonols, vitamins, gluthathione, α-tocopherols, β-carotene, and carotenoids are very good and non-toxic sources of antioxidants [55]. MO has been shown to possess high levels of all of these antioxidant activities [56,57,58]. Increased daily antioxidant consumption can also limit free radical damage. Interestingly, MO is capable of preventing or slowing the oxidation of other molecules generally by trapping free radicals and reducing development of inflammatory cytokines because of its high phenolic contents [59,60]. In this study, antioxidant activities were determined by measuring concentrations of total polyphenol (2454.00 ± 17.54 mg GAE/L), flavonols (297.23 ± 30.00 mg QE/L) and flavonoids (148.70 ± 4.00 mg CE/L) content in methanolic extract of MO (Table 2). High levels of total polyphenols, flavonols and flavonoid content were reported in MO suggesting its ability to lower oxidative stress and reduce cellular damage. This high phenolic content is responsible for its antidiabetic and antioxidant properties.

Antioxidant capacity was used to measure the antioxidant potency and phytochemical concentration of the leaf extract elucidating its free radical scavenging ability [61]. In this study, the antioxidant capacity using oxygen radical absorbance capacity (ORAC), ferric of methanolic MO extract was determined by reducing antioxidant power (FRAP), Trolox equivalence antioxidant capacity (TEAC) of methanolic extract of MO was determined prior to its administration in rats. Results showed ORAC: (3652 ± 113.32 µmol TE/L), FRAP: (1736 ± 3.08 µmol AAE/L), and TEAC: (96.09 ± 1.58 µmol TE/L). This is indicative of radical scavenging capability of MO. The presence of polyphenols revealed phytochemical activity of MO leaves as seen in results.

### 2.3. Determination of Serum Total Protein, Creatinine, Albumin and Globulin Concentrations

In Table 3, total protein and albumin concentrations significantly decreased (*p* < 0.05) in diabetic rats compared to the normal control (non-diabetic control). Creatinine concentration insignificantly increased in diabetic rats and decreased slightly in diabetic and non-diabetic rats after MO treatment. Globulin decreased in diabetic rats but not significantly. Total protein, albumin and globulin levels increased significantly (*p* < 0.05) in MO-treated control rats compared to normal control and diabetic control. In addition, an increase in total protein, albumin and globulin level was observed in diabetic treated rats when compared to diabetic control but with only significant (*p* < 0.05) increase in albumin level when compared to normal control rats.

In a diabetic state, loss in blood protein (albumin and globulin) is observed and, when excessive, alters the normal filtering mechanism of the kidneys resulting in the accumulation of toxic wastes. Albumin and globulin serve as transport proteins and biomarkers in the disease state [62]. In this study, total protein, albumin and globulin levels were lowered in diabetic rats compared to normal control groups while creatinine level slightly increased (Table 3). Creatinine level was also assessed to ascertain the normal function of the kidney. MO administration increased levels of total protein, albumin, and globulin in the serum of rats. Creatinine level reduced in MO treated group suggesting MO ability to enhance and regenerate the kidney functional status after treatment.

### 2.4. Effect of Lipid Peroxidation and Activities of Antioxidant Enzymes in the Kidneys

Table 4 shows the effect of lipid peroxidation and activities of antioxidant enzymes in the kidneys of rats. Diabetic rats showed significantly (*p* < 0.05) increased Malondialdehyde (MDA) levels when compared to normal control. Subsequent treatment of diabetic rats with MO led to a significant (*p* < 0.05) decrease in MDA when compared with non-treated diabetic control. MDA levels decreased in MO-treated control when compared to normal control rats. Activities of catalase (CAT), superoxide dismutase (SOD) and reduced glutathione (GSH) decreased in diabetic rats when compared to normal control and a significant (*p* < 0.05) decrease was observed only in CAT as compared to normal control. MO administration to diabetic rats led to significantly (*p* < 0.05) increased activities of CAT, while SOD and GSH increased but not significantly when compared to diabetic control. CAT, SOD and GSH activities also increased in non-diabetic rats upon MO administration when compared to normal control. Glutathione peroxidase (GPx) activity also decreased significantly in non-diabetic and diabetic treated groups when compared to normal control after treatment with MO.

High concentration of ROS causes damage to structural biomolecules such as proteins, lipids and carbohydrates leading to the inability of the body’s defense mechanism in protecting cellular integrity [63,64]. Free radicals cause lipid peroxidation, and when this happens, the lipids of the cell membrane undergo catabolism, leading to damage of tissues. In this case, polyunsaturated fatty acids of the kidney were broken down and the cell membrane structure was compromised, leading to a disruption in its functionality [65].

In the present study, MDA levels increased significantly in the kidney of diabetic rats when compared to the normal control (Table 4). The increase in MDA level in diabetic groups suggests damage to cell membrane lipids in a diabetic state, which could lead to increased generation of ROS [66]. Subsequent treatment with MO led to a significant (*p* < 0.05) decrease in MDA levels in diabetic rats reducing lipid peroxidation. Similarly, Verma et al. reported reduced MDA levels after treatment of animals with MO, which is in agreement with our results [58]. MO exerts its ameliorative effect by reducing oxidative stress, endothelial cell dysfunction, kidney lipid peroxide and increasing antioxidant capability. This effect may be related to MO’s phytochemical composition, which appears to commence a recovery process from associated kidney related damage.

Antioxidant enzymes (CAT, SOD, GPx) and non-enzymatic antioxidants (GSH) delay or prevent the oxidation of substrates and prevent ROS-induced oxidative stress [67]. SOD represents the first line of defense against oxygen derived radicals (ROS), as it is responsible for the dismutation of superoxide radicals to H_2_O, whereas catalase metabolically removes hydrogen peroxide (H_2_O_2_) and hydroxyl radical generation [8]. The synergistic relationship between CAT and SOD against ROS accumulation inactivates peroxyl radicals and superoxide anions, converting them to water and oxygen [25]. GSH is a thiol group containing molecules, well known for its effective antioxidant property in scavenging hydroxyl radical and singlet oxygen [41]. GPx detoxifies H_2_O_2_ and lipid peroxides using GSH as substrate.

In the current study, the activity of CAT, SOD and GPx were significantly elevated in the kidney tissue homogenate of the diabetic groups (Table 4). GSH reduced in diabetic groups in comparison to non-diabetic control, and treatment did not increase concentration of GSH. Following treatment with MO, the activities of CAT, SOD and GSH were enhanced in all MO treated groups, suggesting MO’s ability to scavenge and neutralize STZ induced oxidative stress, thus providing a significant recovery in the altered enzyme defense mechanism in the treated groups. This may be related to the presence of terpenoids in MO [68].

### 2.5. Effect of Moringa oleifera on Tumour Necrosis Factor (TNF-α) and Interleukin IL-6 in the Kidneys

**Figure 1:**
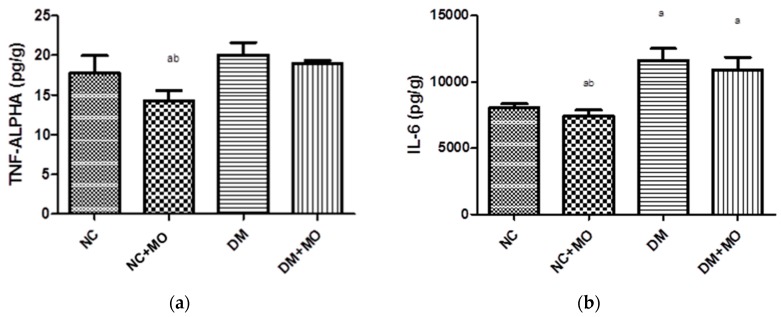
Effect of *Moringa oleifera* on (**a**) tumour necrosis factor-alpha (TNF-α) and (**b**) interleukin-6 (IL-6) in the kidneys. NC (Normal control), NC + MO (*Moringa oleifera*-treated control rats), DM (Diabetic rats) and DM + MO (*Moringa oleifera*-treated diabetic rats). Values are presented as mean (SD). ^a^ *p* < 0.05 values are significant compared with non- diabetic control. ^b^ *p* < 0.05 values are significant compared with diabetic control.

The effect of inflammatory biomarkers TNF-α and IL-6 concentrations in Figure 1a,b showed a gross increase in TNF-α and IL-6 occurring in the diabetic groups when compared to non-diabetic groups. Levels of TNF-α and IL-6 decreased in non-diabetic treated rats when compared to normal control and diabetic control. Administration of MO to diabetic treated rats led to a decreased level of TNF-α and IL-6 when compared to diabetic control.

Inflammation in tissues occurs as a response to harmful stimuli or damage to cells. IL-6 and TNF-α cytokines play a crucial role in hyperglycemia-induced kidney injuries and are associated with the development of diabetes and with increasing levels in patients with diabetes nephropathy, suggesting that these cytokines play significant roles in the pathogenesis of diabetes nephropathy as reported by Navarro et al. [50]. Seca et al. demonstrated, in their study, the presence of a high concentration of inflammatory proteins, leading to the destruction of insulin produced by the pancreatic beta cells [68].

**Figure 2:**
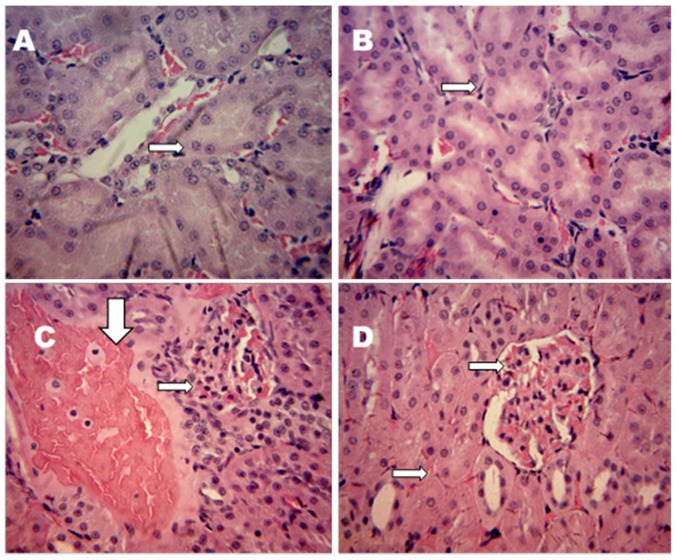
Effect of *Moringa oleifera* on the kidney sections (hematoxylin and eosin stained X 400). (**A**) NC (Normal control) (**B**) NC + MO (*Moringa oleifera*-treated control rats) (**C**) DM (Diabetic rats) and (**D**) DM + MO (*Moringa oleifera*-treated diabetic rats).

There is convincing experimental and clinical evidence, which indicates an increase in the generation of ROS and systemic markers of inflammation in both types of diabetes, which is in line with our study [50,69]. Diabetic-induced rats (Figure 2) showed an increased level of IL-6 and TNF-α, which reduced with concurrent treatment with MO, which shows MO as a potent anti-inflammatory agent. Our results are consistent with the findings of Al-Malki et al. who reported a decrease in inflammatory cytokines after treatment with MO [30]. The observed decrease in the inflammatory cytokines shows retardation in the onset of diabetic nephropathy [70,71].

In this study, treatment with MO almost restored to normal the adverse effects caused by STZ in the kidneys after a period of six weeks. The results showed that MO extracts have the potency to counteract the formation of free radicals and other reactive species generated by the disease state in the kidneys, thereby decreasing oxidative stress and preventing oxidative damage.

### 2.6. Effect of Moringa oleifera on Kidney Histopathology Annotations

Histopathological examination of the kidney sections of non-diabetic and diabetic rats revealed the protective effect of MO on the kidneys (Figure 2A–D). Non-diabetic control and non-diabetic treated rats showed no visible lesions and normal glomeruli (Figure 2A,B). In diabetic control rats, severe interstitial congestion at the cortical area of the kidney was observed. Haemorrhage and congestion of the glomerulus were also observed (Figure 2C). Diabetic treated rats showed very mild vascular congestion of the glomerulus (Figure 2D).

Microscopic anatomical analysis of the kidney tissue was performed (Figure 2A–D). Histopathological sections of the kidney of non-diabetic rats indicated normal cell structure. Kidney sections of diabetic rats demonstrated severe renal damage showing interstitial nephritis at the cortical area of the kidney. Glomeruli haemorrhage was associated with diabetic rats. However, administration of MO showed appreciable improvements to these alterations with mild vascular congestion of the glomerulus. This result suggests that diabetic nephropathy was significantly reduced after MO treatment. This is consistent with other findings who reported significant changes in the pathology of the kidney of diabetic rats after treatment [5,72].

## 3. Materials and Methods

### 3.1. Chemicals

Streptozotocin (STZ), quercetin, 6-hydroxydopamine, 6-hydroxy-2,5,7,8-tetramethylchroman-2-carboxylic acid (Trolox) and 2-thiobarbituric acid (TBA) and β-nicotinamide adenine dinucleotide phosphate reduced tetra-sodium salt (NADPH) were obtained from Sigma-Aldrich (Johannesburg, South Africa). Methanol, malondialdehyde bis (diethyl acetal) (MDA), *n*-hexane were purchased from Merck (Johannesburg, South Africa). Other chemicals were of the highest grade and procured from Sigma-Aldrich (St. Louis, MO, USA) and Merck (Darmstadt, Germany).

### 3.2. Collection of Plant Material

Fresh MO leaves and flower were obtained from botanical garden of the Forestry Research Institute of Nigeria (FRIN), Ibadan, Nigeria in October 2014. The plant was authenticated by a plant taxonomist, Mr. A.A Adeyemo, with a voucher specimen (FHI-110287) deposited in the Institute’s herbarium.

#### Extract Preparation

Green leaves of MO were washed, air dried and blended to powdery form. Extract was prepared from 1 kg of MO powder via continuous stirring in n-hexane for 24 h. The residue was re-extracted in 80% (*v*/*v*) methanol (Merck, Johannesburg, South Africa) at room temperature for 24 h. The methanolic extract was evaporated to dryness in vacuo using rotary evaporator (Heidolph Instruments, Schwabach, Germany) and stored at −4 °C for use and phytochemical screening.

### 3.3. Ethical Statement

All experimental protocols described in this study was approved by the Faculty of Health and Wellness Sciences, Research Ethics Committee (REC) of Cape Peninsula University of Technology (CPUT), Bellville, South Africa with REC approval reference number: CPUT/HW-REC 2014/AO8. All animals received humane care according to the principles of laboratory animal care of the National Institutes of Health Guide for the care and use of laboratory animals of the National Academy of Science (NAS) publication No. 80–23, revised 1978.

### 3.4. Study Design

#### 3.4.1. Experimental Animals

Experimental animals used for this study were adult male Wistar rats weighing about 200 g and 250 g, and aged 10 weeks. The source of the animal is the Stellenbosch Animal facility, Tygerberg, South Africa. The animals were housed in the same animal facility and used for the experiment. Forty-eight rats were randomly divided into 4 groups. Rats were housed in a well-ventilated animal facility in stainless steel cages (beddings composed of ground sterilized maize cobs) with 5 rats per cage to allow free mobility. Rats were fed with standard rat chow (Aquanutro, Mamelsbury, South Africa) and water ad libitum. Conducive temperature of 22 ± 2 °C, humidity 55% ± 5% and a normal period (12 h light/12 h dark) was maintained.

#### 3.4.2. Induction of Diabetes

A diabetic state was induced in the rats by injecting intraperitoneally (i.p.) freshly prepared streptozotocin (STZ, Sigma, St. Louis, MA, USA) in citrate buffer (0.1 M pH 4.5) to overnight fasted rats at a dose of 55 mg/kg [6]. Blood was obtained from the rat’s tail to confirm a diabetic state using a glucometer (Accu-Check, Roche, Manheim, Germany). A stable glucose level of (>18 mmol/L) confirmed hyperglycemia, and only diabetic rats were included in the study.

#### 3.4.3. Treatment

A dose of *Moringa oleifera* (250 mg/kg) is the most suitable based on preliminary investigations in our research centre. Forty-eight rats were randomly divided into four groups of twelve rats each; NC–Normal non-treated control, NC + MO—*Moringa oleifera* treated control rats, DM—diabetic rats and DM + MO—*Moringa oleifera* treated diabetic rats. NC and DM (control groups) received distilled water while MO and DM + MO (experimental groups) received *Moringa oleifera* extract at a dose of (250 mg/kg/b.wt.). Distilled water was used as the diluent for reconstructing the extract and administered via oral gavage for 6 weeks.

At the end of the treatment, rats were fasted overnight and anaesthetized intraperitoneally with sodium pentobarbital injection (60 mg/kg). Sodium pentobarbital was used to ensure unconsciousness of rats while death occurred as well as guaranteeing rapid and painless death. This procedure was carried out in the animal house. Blood samples were obtained via the rat’s abdominal aorta into a lithium heparin plasma separator tubes and serum clot activator tubes. The whole kidneys were quickly excised from each rat, washed in ice-cold phosphate-buffered saline, blotted, weighed and frozen in liquid nitrogen.

#### 3.4.4. Blood and Homogenate Preparation

Blood samples were centrifuged at 4000 g for 10 min at 4 °C and then stored at −80 °C to obtain plasma and serum. Kidneys (200 mg) were homogenized on ice in 2000 µL ice-cold phosphate buffer saline (PBS, 50 mM pH 7.5). Homogenates were centrifuged at 15,000 rpm for 10 min at 4 °C. The supernatants were aliquoted and stored at −80 °C for estimation of biochemical parameters.

### 3.5. Experimental Analysis

#### 3.5.1. Relative Kidney Weight

The relative kidney weight was estimated by comparing the kidney weight to the body weight of the same rat:

$$Relative\;Kidney\;weight\;\left(mg/100\;g\;body\;weight\right)=\;\frac{Kidney\;weight\;\left(g\right)}{Total\;body\;weight\;\left(g\right)}\;\times 100$$

#### 3.5.2. Plasma Glucose Determination

Plasma collected from blood was used for determination of plasma glucose level using a Randox kit from Randox Laboratories Limited (Crumlin, UK). Standard protocol was followed according to the manufacturer’s operating procedures.

### 3.6. Antioxidant Capacity of Moringa oleifera Methanolic Extracts

#### 3.6.1. Oxygen Radical Absorbance Capacity (ORAC)

Antioxidant capacity was performed on the extract; oxygen radical absorbance capacity (ORAC) in MO extracts was analyzed using Ou’s method [73]. The reaction measures the capability of the extract to scavenge free radicals. A fluoroscan plate reader (Thermo Fisher Scientific, Waltham, MA, USA) with a 96-well plate reader was used to monitor reaction at 485 nm and 538 nm wavelengths for a two-hour ORAC assay was carried out by addition of 138 µL working fluorescein, 12 µL of Trolox used as the reference antioxidant, 12 µL of extracts, followed by 50 µL 2,2′-azobis (2-amidinopropane) dihydrochloride (AAPH), which generates peroxyl radicals that initiated the onset of the reaction. Values were expressed as µmol Trolox equivalent per gram dry weight (TE/g DW).

#### 3.6.2. Ferric Reducing Antioxidant Power (FRAP)

FRAP was assessed to determine the total antioxidant power of MO using the protocol adapted from Benzie and Strain [74]. In addition, 10 μL of standard (l-Ascorbic acid) was added followed by the addition of 10 μL of extract, 300 μL of FRAP reagent, which included (10 mM 2,4,6-tripyridyl-s-triazine (TPTZ), 300 mM acetate buffer at pH 3.6 (Saarchem, Krugersdorp, South Africa) in 0.1 M HCl, 20 mM Iron (III) Chloride hexahydrate (FeCl_3_·6H_2_O) (Sigma, Johannesburg, South Africa) and 6.6 mL distilled water, which produced a straw colour. All reagents were incubated for 30 min before the reading was taken. Absorbance was measured at 593 nm. The results were expressed as μM ascorbic acid equivalent per g dry weight (μM AAE/g DW).

#### 3.6.3. Trolox Equivalence Antioxidant Capacity (TEAC)

TEAC measures the antioxidant capacity of MO, as compared to the standard, Trolox. TEAC assay was carried out using the method described by Re et al., a stock solution was prepared from 2,2′-azino-bis (3-ethylbenzothiazoline-6-sulphonic acid) ABTS (8 mM) and potassium persulfate K_2_S_2_O_8_ (3 mM) formed ABTS^+^, which was prepared 24 h before the analysis was performed. Trolox was used as the standard, 25 μL of extract and 275 μL of ABTS mix was added to the well. A Multiskan plate reader (Thermo Electron Corporation, Beverly, MA, USA) was used to read absorbance at 734 nm. Results were expressed as µmol TE/L [75].

### 3.7. Phytochemical Investigation: Total Polyphenol, Flavonoids and Flavonols Content of Moringa oleifera Extract

Total polyphenol content was determined in extracts of MO using a Folin–Ciocalteu protocol, Folin’s reagent; sodium carbonate and extract were added to wells with Gallic acid as the standard. A Multiskan plate reader (Thermo Electron Corporation, Beverly, MA, USA) was used to read absorbance at 765 nm. Results were expressed as mg GAE/g dry weight [76].

Total flavonoid content was determined in MO extract by a colorimetric method described by Singleton. Results were expressed as catechin standard equivalent mg CE per gram extract and absorbance read at 510 nm [76]. Total flavonols content was extracted with (2% HCl, 95% ethanol and quercetin as standard). Results were measured spectrophotometrically at 360 nm and expressed as mg/QE/g dry weight [77].

### 3.8. Serum Total Protein, Creatinine, Albumin and Globulin Determination

Total protein concentration was estimated in the serum with a Randox kit using an automated Randox Daytona analyzer from Randox Laboratories Limited. A coloured complex was formed through a reaction that occurs between protein and cupric ions in alkaline medium. All protocols were in accordance to the manufacturer’s instruction.

Creatinine concentration was measured in the serum using Randox kit on the automated Randox Daytona analyzer from Randox Laboratories Limited. A red-orange coloured complex was formed through the reaction of creatinine with picrate ion. All protocols were followed according to the manufacturer’s instructions.

Serum albumin level was determined using kits from Randox Laboratories Limited. The principle is based on its ability to bind to 3,3′,5,5′-tetrabromo-m cresol sulphonphthalein. The complex formed was measured at 578 nm.

Globulin level was determined using the formula:

Globulin =Total protein –Albumin.

### 3.9. Lipid Peroxidation and Activities of Antioxidant Enzymes in the Kidneys 

Lipid peroxidation was determined in the kidney homogenate by measuring the conjugated dienes and malondialdehyde (MDA) using high performance liquid chromatography-HPLC. (Agilent 1200 Technologies, Bellefonte, PA, USA) with a diode array detector, 5 μm column C18 (4.6 mm × 150 mm). Furthermore, 50/50 methanol was used as the mobile phase, run rate at 10 min and 1mL/min flow rate. Fluorometric detection was performed with excitation at 532 nm and emission at 552 nm. The peak of the MDA-TBA adduct was calibrated with the MDA standard. MDA was used as the standard and results expressed as µmol MDA/g for kidney homogenates. MDA, an end product of lipid peroxidation, was determined using Khoschsorur’s method [78].

Superoxide Dismutase (SOD): Activity of SOD was assessed using a 96-well Multiskan plate Thermo Electron Corporation reader (Beverly, MA, USA) modified for a microplate reader at 490 nm and expressed as the amount of protein [mg] required to produce a 50% inhibition of auto-oxidation of 6-hydroxydopamine [79].

Catalase (CAT): Activity of CAT was determined spectrophotometrically at 240 nm. This was done by monitoring the decomposition of H_2_O_2_ and expressed as μmole H_2_O_2_/µg protein [80].

Total glutathione (GSH): The level of total glutathione GSH in the kidney was estimated [81]. All of the analyses were done using a 96-well plate and read with a Multiskan Spectrum plate reader (Thermo Fisher Scientific, USA).

Glutathione Peroxidase (GPx): Level of GPx activity in the kidney was determined spectrophotometrically at 340 nm and expressed as μmoles NADPH/ mg protein [82].

### 3.10. Determination of Tumour Necrotic Factor (TNF-α) and Interleukin (IL-6) in the Kidney

Levels of inflammatory biomarkers, tumour necrotic factor (TNF-α) and interleukin (IL-6) in the kidney were determined using Millipore’s MILLIPLEX^®^ MAP rat cytokine magnetic bead panel kit (Millipore Cooperation, Billerica, MA, USA). The protocol was followed according to the manufacturer’s instruction. The assay was performed on the Bio-plex^®^ platform (Bio-Rad, Hercules, CA, USA) and Bio-Plex Manager TM software (version 6.0, Hercules, Hercules, CA, USA) was used for analysis.

### 3.11. Kidney Histopathology

Histopathology of the kidney tissues was performed. The kidney tissue was fixed in buffered formalin (10%) and then dehydrated in ethanol, after which it was embedded in paraffin using a Leica EG1160 embedder (Leica Microsystems, Inc., Buffalo Grove, IL, USA). The tissue blocks were cut to 5 µm with a Leica microtome (Leica RM2125, Leica Microsystems, Inc.) and hematoxylin and eosin stained for histological examination. The slides were examined under a light microscope at a magnification of ×400.

### 3.12. Statistical Analysis

The values were analyzed using Prisms Graph pad 5.0 (Graph Pad software for Windows, San Diego, CA, USA) to calculate the mean ± SD (standard deviation). One-way analysis of variance (ANOVA) was used to compare differences in multiple groups with Newman–Keuls Multiple Comparison as a post hoc test. *p* < 0.05 was considered statistically significant between control and test means.

## 4. Conclusions

Based on the experimental evidence from our study, results suggest that *Moringa oleifera* has an excellent ability to protect against oxidative damage due to its high polyphenols, flavonoids and flavonols content. Its use as a food supplement can be justified due to its therapeutic benefits. Hyperglycemia was successfully induced in the animal model with STZ, which was confirmed in the blood and kidney biomarkers. Oxidative stress was observed in diabetic groups while treatment with a methanolic extract of MO ameliorated the effect. From our study, MO was also able to enhance antioxidant status and reduce lipid peroxidation, showing that MO has the potential to be used as an antidiabetic agent in the treatment and management of diabetes.

Further studies are recommended in isolating phenolic compounds in pure form so as to establish the mechanisms of action and the structural activity of *Moringa oleifera.*

## Figures and Tables

**Table 1:** Effect of *Moringa oleifera* on kidney weight, relative kidney weight and plasma glucose levels.

	NC	NC + MO	DM	DM + MO
Kidney weight (g)	1.90 ± 0.17	1.80 ± 0.17 ^b^	2.38 ± 0.18 ^a^	2.13 ± 0.20 ^a,b^
Relative kidney weight (g/100 g)	0.60 ± 0.04	0.57 ± 0.03 ^b^	1.10 ± 0.08 ^a^	1.03 ± 0.08 ^a,b^
Plasma glucose (mmol/L)	8.42 ± 0.82	5.01 ± 0.53 ^a,b^	28.08 ± 1.12 ^a^	26.22 ± 0.61 ^a,b^

NC (Normal control), NC + MO (*Moringa oleifera*-treated control rats), DM (Diabetic rats), DM + MO (*Moringa oleifera*-treated diabetic rats). Values are presented as mean (SD). ^a^ *p* < 0.05 values are significant compared with non-diabetic control. ^b^ *p* < 0.05 values are significant compared with diabetic control.

**Table 2:** Estimation of antioxidant capacity, total polyphenols, flavonoids and flavonols content of *Moringa oleifera* extracts.

MO Methanolic Extract	
ORAC (µmol TE/L)	3652.14 ± 113.32
FRAP (µmol AAE/L)	1736 ± 3.08
TEAC (µmol TE/L)	96.09 ± 1.58
Total polyphenol (mg GAE/L)	2454.00 ± 17.54
Flavonol (mg QE/L)	297.23 ± 30.00
Flavonoid (mg CE/L)	148.70 ± 4.00

Values are presented as (mean ± SD). TE (Trolox equivalent); AAE (ascorbic acid equivalent); CE (catechin equivalent); GAE (Gallic acid equivalent; QE (quercetin equivalent).

**Table 3:** Serum total protein, albumin and globulin concentrations.

Parameters	NC	NC + MO	DM	DM + MO
**Total protein (g/L)**	53.86 ± 2.36	70.04 ± 6.05 ^a,b^	44.66 ± 6.92 ^a^	50.54 ± 1.76
**Creatinine (g/L)**	50.21 ± 0.81	49.31 ± 3.01	52.23 ± 2.05	51.06 ± 1.00
**Albumin (g/L)**	32.50 ± 0.89	39.36 ± 3.03 ^a,b^	27.55 ± 1.17 ^a^	29.69 ± 0.94 ^a^
**Globulin (g/L)**	22.98 ± 2.13	43.34 ± 3.89 ^a,b^	21.21 ± 0.79	26.06 ± 7.68

NC (Normal control), NC + MO (*Moringa oleifera*-treated control rats), DM (Diabetic rats), DM + MO (*Moringa oleifera*-treated diabetic rats). Values are presented as mean (SD). ^a^ *p* < 0.05 values are significant compared with non-diabetic control; ^b^ *p* < 0.05 values are significant compared with diabetic control.

**Table 4:** Lipid peroxidation and activities of antioxidant enzymes in the kidney.

	NC	NC + MO	DM	DM + MO
MDA (µmol/g)	0.48 ± 0.04	0.43 ± 0.05 ^b^	0.72 ± 0.12 ^a^	0.54 ± 0.06 ^b^
CAT (U/mg protein)	0.42 ± 0.02	0.47 ± 0.01 ^b^	0.28 ± 0.06 ^a^	0.30 ± 0.05 ^ab^
SOD (U/mg protein)	1.01 ± 0.40	1.35 ± 0.11 ^ab^	0.78 ± 0.30	0.92 ± 0.0.1
GSHt (µmol/g)	2.00 ± 0.26	1.70 ± 0.28	1.67 ± 0.47	1.86 ± 0.0.24
GPx (U/mg protein)	1.29 ± 0.33	0.72 ± 0.23 ^a^	1.11 ± 0.40	0.87 ± 0.40 ^a^

NC (Normal control), NC + MO (*Moringa oleifera*-treated control rats), DM (Diabetic rats), DM + MO (*Moringa oleifera*-treated diabetic rats). Values are presented as mean (SD). ^a^ *p* < 0.05 values are significant compared with non-diabetic control; ^b^ *p* < 0.05 values are significant compared with diabetic control.
